# Imagining the Future Aged Self Reduces Ageism: The Role of Self–Other Overlap and the Moderating Effect of Gain–Loss Framing

**DOI:** 10.3390/bs16050783

**Published:** 2026-05-15

**Authors:** Dexian He, Quan He, Hongyan Zhu, Xianyou He

**Affiliations:** 1 Department of Psychology, Guangdong University of Education, Guangzhou 510303, China; dexianhe@m.scnu.edu.cn; 2 Guangdong Key Laboratory of Mental Health and Cognitive Science, South China Normal University, Guangzhou 510631, China; 3 Center for Studies of Psychological Application, South China Normal University, Guangzhou 510631, China; 4 School of Psychology, South China Normal University, Guangzhou 510631, China; 5 School of Integrative Medicine, Nanchang Medical College, Nanchang 330000, China

**Keywords:** ageism, future-aged-self perspective taking, self-other overlap, prosocial behavior, gain-loss framing

## Abstract

Population aging poses growing social and economic challenges, yet effective psychological interventions targeting ageism remain limited. The present research examined whether future-aged-self perspective taking increases self–other overlap with older adults and promotes prosocial behavioral responses toward them, and whether these effects depend on decision-making context. In Study 1 (*N* = 160), participants completed a perspective-taking task followed by a Dictator Game. Individuals who imagined their future aged self reported greater self–other overlap with older adults and allocated more resources to older, compared with younger, targets. Study 2 (*N* = 143) extended this investigation using a Welfare Trade-Off Task that manipulated gain versus loss framing. Participants in the future-aged-self condition again reported higher self–other overlap and stronger intentions to communicate with older adults. They also showed higher welfare trade-off ratios favoring older adults under gain-framed conditions, whereas no significant group difference emerged under loss framing. These findings suggest that future-aged-self perspective taking can enhance young adults’ prosocial responses toward older adults, but that its effectiveness is contingent on situational framing. Temporal-self interventions may be most effective when prosocial action is framed as allocating potential gains rather than accepting explicit personal losses.

## 1. Introduction

### 1.1. Ageism: Scope, Manifestations, and Consequences

Population aging is a defining trend. By 2050, the global population of older adults is expected to reach around 2.1 billion (World Health Organization, 2025). Despite this dramatic demographic shift, older people continue to experience implicit and explicit age-based bias in everyday life, work, and healthcare. Ageism is not only an intergroup bias but also a temporal self-bias: the very group derogated today is the self one will become tomorrow. Compared with sexism and racism, research on ageism has been relatively sparse and effective interventions remain limited (North & Fiske, 2012). The present research examines whether imagining one’s future aged self increases self–other overlap, that is, the perceived psychological closeness and cognitive overlap between the self and older adults, and, in turn, promotes prosocial behavior toward older adults. Importantly, we extend prior work by testing whether such effects generalize from relatively low-cost, gain-framed contexts, in which helping mainly involves giving up potential benefits rather than incurring explicit personal losses, to situations in which helping entails potential personal costs. We examine these effects using behavioral welfare-allocation measures, namely decision tasks that assess how participants allocate monetary outcomes between themselves and older adults, rather than relying on attitudes alone.

Stereotypes about older adults are multidimensional. Within the framework of the Stereotype Content Model, older adults are typically perceived as warm but less competent (Cuddy & Fiske, 2002; Cuddy et al., 2007). Compared with other age groups, they are often viewed as trustworthy, kind, and well-intentioned, yet simultaneously perceived as frail, less capable, and resistant to change (Kite et al., 2005; North & Fiske, 2015; Spaccatini et al., 2022). Their cognitive abilities, work efficiency, and social skills are generally regarded as declining, and they are sometimes portrayed as a burden on economic and healthcare systems (Ayalon, 2020; Sutter et al., 2022). Moreover, people tend to process older adult faces primarily in terms of categorical membership rather than individual characteristics (He et al., 2021).

These prejudicial attitudes have wide-ranging practical ramifications (Lytle & Levy, 2019; North & Fiske, 2012). In interpersonal contexts, people show decreased willingness to communicate or cooperate with older adults and may neglect their autonomy in decision-making (Shepherd & Brochu, 2021; Wang et al., 2014). In the workplace, older employees are perceived as less competent and less suitable for promotion (Chang et al., 2020; North & Fiske, 2016), and in healthcare, older patients may be denied equitable access to medical services (Christian et al., 2014; Cuddy et al., 2005). Furthermore, Levy’s (2009) Stereotype Embodiment Theory (SET) posits that internalized negative stereotypes can have deleterious effects on older adults’ mental, physical, and emotional well-being, contributing to depression, loneliness, cognitive decline, and even reduced longevity (Dionigi, 2015; Levy, 2003; North & Fiske, 2012).

### 1.2. The Temporal Nature of Ageism and Future-Self Perspective Taking

Ageism is a form of intergroup bias, a systematic tendency to favor one’s ingroup over an outgroup (Riek et al., 2006; Oh et al., 2016). According to Social Identity Theory (SIT; Tajfel & Turner, 1979, 1986), individuals derive part of their self-concept from social group membership and strive to maintain a positive social identity. This motivation often manifests as ingroup favoritism and outgroup derogation, suggesting that ageism may function as a means for younger adults to protect their self-esteem (Bodner, 2009; Kite & Wagner, 2002). Social categorization shapes how people think, feel, and behave toward ingroup and outgroup members. Empirical evidence shows that when older adults are categorized as outgroup members, younger adults show more hostile ageism and less helping behavior toward them (Chen & Zhang, 2022; G. Ma et al., 2024; Tasdemir, 2020). People with higher youth identity also tend to exhibit stronger ageist attitudes, possibly because they see older adults as a symbolic threat to their vitality and identity (Shimizu & Grassi, 2022; Shimizu et al., 2022). This “us versus them” mindset may foster a zero-sum mentality that saps support for public policies benefiting older adults (Monahan et al., 2020).

Unlike sexism or racism, ageism targets both “others” and one’s future self, creating a unique opportunity for self-based interventions that link present and future identities. Aging is an inevitable developmental process, meaning that individuals are not merely observers but also eventual participants of the aging process (Levy, 2009; North & Fiske, 2012). This temporal nature offers a conceptual leverage point for interventions that emphasize the connection between present and future selves. When people realize that “becoming old” is not distant, the categorical boundary between “us” and “them” may soften, thereby reducing negative bias (Shimizu & Grassi, 2022; Shimizu et al., 2022).

Recent interventions grounded in Stereotype Embodiment Theory (SET) demonstrate this possibility. Shimizu et al. (2022) found that presenting participants with the core ideas of SET and empirical evidence about stereotype internalization shortened their subjective time to becoming older and reduced anti-old attitudes. Similarly, Wen et al. (2022) reported that shifting group identity effectively alleviated intergroup bias and enhanced explicit impressions of older adults. Taken together, these findings suggest that when individuals perceive older adults as part of their ingroup rather than an outgroup, negative attitudes and behaviors toward this group can be reduced. Such perception can be cultivated through perspective-taking strategies that encourage people to imagine their future aged selves, thereby increasing the level of self–other overlap.

Self–other overlap refers to the extent to which individuals include others in the self by incorporating others’ resources, perspectives, and traits into their own self-concept, resulting in partially overlapping cognitive representations of self and other (Aron et al., 2013). A higher level of overlap enables individuals to better understand others’ intentions and emotional states (Bernstein et al., 2015) and strengthens interpersonal bonds (Thai & Lockwood, 2015). In intergroup contexts, greater self–other overlap has been associated with reduced prejudice, more differentiated perceptions of outgroups, more favorable trait attributions, stronger willingness for intergroup contact, and greater prosocial behavior toward outgroup members (Gaesser & Schacter, 2014; Galinsky & Moskowitz, 2000; Hewstone et al., 2014; Stathi & Crisp, 2008; Turner et al., 2007; Wang et al., 2014; Zhong et al., 2015). Perspective taking is one commonly used strategy for increasing self–other overlap because it encourages individuals to adopt another person’s standpoint and simulate that person’s experiences.

Several studies have employed embodied or simulated aging paradigms to reduce bias. For instance, Green and Dorr (2016) found that simulated aging activities (e.g., vision and mobility impairments) led to more positive attitudes and stronger helping intentions toward older adults. Oh et al. (2016) used virtual perspective taking by having participants embody an aged avatar in VR, which increased willingness to interact with older adults by strengthening self–other overlap. Chen and Zhang (2022) further showed that imagining their future selves reduced ageist attitudes, even under intergenerational resource competition. Similarly, Z. Liu et al. (2023) developed EmpathiaVR, a multisensory virtual reality system simulating older adults’ visual, auditory, and kinesthetic experiences, which significantly enhanced users’ empathy. However, research directly examining how increasing self–other overlap with older adults can reduce age-related prejudice remains limited and warrants further investigation. In addition, prior work has rarely combined future-aged-self perspective taking with explicit measures of welfare allocation, leaving open the question of whether such manipulations can shift how younger adults distribute concrete resources between themselves and older adults.

Moreover, imagining the future self has shown broad effects on moral and prosocial behavior. Bartels and Rips (2010) proposed that the degree of psychological connectedness between the current and future self is a critical determinant of intertemporal choices; when this connectedness is strong, individuals treat their future selves with the same concern as their present selves. Building on this, Hershfield et al. (2012) demonstrated that people with greater future self-continuity, a sense of connectedness between their present and future selves, were more ethical, generous, and patient in decision-making. Van Gelder et al. (2013) provided a compelling demonstration using immersive virtual reality: participants interacted with an age-progressed digital rendering of themselves and subsequently engaged in fewer dishonest behaviors than those who saw their current selves, suggesting that visualizing one’s aged self heightens responsibility toward the future self. Similarly, Engle-Friedman et al. (2022) found that participants who imagined, drew, and described themselves at age 60 behaved more sustainably in a simulated resource-management task than those who imagined themselves in the present. Even brief future-thinking inductions have been shown to promote helping behavior (Curotto et al., 2022). Recently, Law et al. (2025) further demonstrated that personal future-thinking enhances intergenerational responsibility and long-term societal concern.

Taken together, these studies suggest that visualizing one’s future or aged self can increase self–other overlap, reducing the perceived boundary between the present and future self. When the future self feels psychologically close, individuals tend to act more responsibly, showing greater moral restraint and prosocial engagement (Bartels & Rips, 2010; Simić et al., 2025). This heightened connectedness may encourage prosocial and ethical decisions by making the consequences for one’s future self more psychologically salient. Extending this logic to ageism, we propose:

**Hypothesis** **1:** *Engaging in a perspective-taking task that involves imagining one’s future aged self will increase self–other overlap with older adults and enhance ageism-related prosocial behavior toward this group.*

Study 1 provides an initial test of this hypothesis using a Dictator Game allocation paradigm, a relatively low-cost prosocial decision context in which participants decide how much to allocate to older versus younger targets, primarily by forgoing potential gains rather than incurring explicit personal losses. This design allowed us to examine whether future-aged-self perspective taking shifts resource allocation toward older adults.

### 1.3. Welfare Trade-Offs and Contextual Moderation: Gain Versus Loss

While imagining one’s future aged self can foster self–other overlap and promote prosocial attitudes toward older adults, such effects may be context-dependent. In everyday social life, individuals often face situations where resources are finite and benefits to one group imply losses to another. According to Realistic Group Conflict Theory (Sherif, 1966), competition over limited and valuable resources such as employment, healthcare, or social welfare naturally evokes a zero-sum mindset in which one group’s gain is perceived as another’s loss. North and Fiske (2013) argued that younger adults are particularly likely to endorse prescriptive stereotypes (e.g., “older people should step aside or limit consumption”) about older people because they often perceive themselves as resource-disadvantaged and thus stand to gain from older adults’ withdrawal. Empirical work supports the view that resource threats increase ingroup favoritism, reduce prosocial behavior, and fuel prejudice or even hostility toward competing groups (Riek et al., 2006; Wlodarczyk et al., 2014). Consequently, although the prosocial effects of future-self or perspective-taking interventions have often been examined in relatively cooperative, low-conflict, or gain-framed contexts, it remains unclear whether such effects extend to conditions involving perceived loss or resource competition.

Building on this, prior research on perspective taking provides valuable insight into how contextual factors shape prosociality. Numerous studies have shown that perspective taking can promote prosocial behavior toward others, including out-group or stigmatized targets (Delton & Robertson, 2016; Kirkpatrick et al., 2015; McCullough et al., 2013). However, most of these studies have been conducted in non-conflictual or gain-framed contexts. As Sassenrath et al. (2022) emphasize, the prosocial effects of perspective taking are highly context-dependent. Real-life decisions often involve trade-offs under conditions of resource scarcity or potential loss, requiring individuals to determine whether they or others should bear the greater cost, a situation that can be described as choosing “the lesser of two evils” (Gao et al., 2020). Decision-making theories such as Prospect Theory (Kahneman & Tversky, 1979) and the Welfare Trade-off Ratio model (Delton & Robertson, 2016) predict that behavior in loss contexts differs systematically from that in gain contexts, due to loss aversion and asymmetrical valuation of outcomes.

Empirical evidence supports this asymmetry. Feng et al. (2021) and Luo et al. (2024) showed that empathy and fairness motives influence prosociality differently in gain versus loss frames, while Li et al. (2020) found that the effect of social distance on prosocial behavior was present in gain contexts but absent in loss contexts. Similarly, Ortiz-Riomalo et al. (2021) found that inducing perspective taking among real resource users produced weaker or more strategic cooperation when actual resource conflict was present. These findings indicate that while perspective taking is often effective in gain-framed or non-conflict situations, its influence may diminish or qualitatively change in loss or high-conflict contexts. Nevertheless, research directly testing perspective-taking effects under loss frames remains scarce. Our study addresses this limitation by directly contrasting gain- and loss-framed welfare trade-offs, allowing us to determine whether the benefits of future-aged-self perspective taking extend beyond low-cost contexts and into decisions involving potential personal losses.

One of the most fundamental questions in social decision-making concerns how individuals balance their own welfare against that of others (Rilling & Sanfey, 2011; Su et al., 2012). The welfare trade-off ratio (WTR) captures the degree to which an individual is willing to sacrifice personal benefits to increase the welfare of another person (Delton, 2010). Tooby et al. (2008) proposed that the human brain has evolved a computational mechanism that calculates trade-offs between self and other interests, formalized as the welfare trade-off ratio (WTR = x_j_/x_i_, where x_j_ represents the benefit to another person, and x_i_ represents the benefit to oneself). A higher WTR indicates a greater willingness to incur costs for others’ welfare, reflecting stronger prosocial or altruistic tendencies, whereas a lower WTR suggests more self-interested behavior. The WTR is typically measured using the Welfare Trade-Off Task (WTT; Delton, 2010; Kirkpatrick et al., 2015), in which participants make a series of choices between monetary outcomes for themselves and another person.

Prior research indicates that people’s WTR decline when interacting with socially distant or threatening outgroups (Delton & Robertson, 2016; Hall et al., 2021; Kirkpatrick et al., 2015) and vary systematically with psychological distance and gain–loss framing (Gao et al., 2020). Integrating this approach with perspective-taking paradigms therefore provides a useful way to quantify cognitive and motivational processes relevant to ageism-related decision-making. Specifically, it allows us to assess whether imagining one’s future aged self can elevate WTR toward older adults, and whether such effects differ across gain and loss contexts. By extending the WTT to intergenerational decision-making, the present research provides a methodological extension and a theoretical contribution to understanding how welfare trade-offs, perspective taking, and ageism-related prosocial decision-making are linked.

Although a higher level of self–other overlap promotes altruistic and prosocial behavior, this effect may diminish under conditions of loss framing. According to realistic group conflict theory (Sherif, 1966) and prospect theory (Kahneman & Tversky, 1979), individuals exhibit stronger loss aversion and self-interested motivations in such contexts. Prior studies have also shown that the prosocial effects of perspective taking are highly context-dependent. They tend to emerge more readily in low-cost and noncompetitive situations but weaken in high-conflict or loss scenarios (Delton & Robertson, 2016; Gao et al., 2020; Hall et al., 2021; Kirkpatrick et al., 2015). We therefore propose:

**Hypothesis** **2:** *The prosocial effects of imagining one’s future aged self will enhance young adults’ welfare trade-off ratios (WTR) toward older adults and promote ageism-related prosocial responding.*

However, this effect will be moderated by prosocial context. It will be stronger in gain-framed scenarios and substantially weaker or absent in loss-framed scenarios, reflecting a Group × Scenario interaction.

## 2. Study 1

### 2.1. Materials and Methods

#### 2.1.1. Participants

A total of 160 participants were recruited from South China Normal University and Guangdong University of Education (88 females; age: *M* = 22.07, *SD* = 3.31, range: 18–33 years; education: *M* = 15.75, *SD* = 2.28). Sample size was determined using G*Power 3.1.9.6, following Dreber et al. (2013) and Kirkpatrick et al. (2015). With a medium effect size (f = 0.25), α = 0.05, and power (1 − β) = 0.95, the minimum required sample size was estimated to be *N* = 54. The final sample therefore substantially exceeded the minimum requirement. Participants were randomly assigned to one of two groups: (1) Future-aged-self condition, *n* = 80, 43 females; age: *M* = 22.02, *SD* = 3.26, range: 18–31 years; education: *M* = 15.68, *SD* = 2.21. (2) Current-self condition, *n* = 80, 45 females; age: *M* = 22.11, *SD* = 3.39, range: 18–33 years; education: *M* = 15.82, *SD* = 2.37. All participants provided written informed consent and received 20 RMB as compensation. The study protocol was approved by the Institutional Review Board of Guangdong University of Education. Data collection was conducted between September and December 2024.

#### 2.1.2. Materials

The stimulus set used in the present study comprised 30 face images, including 15 younger faces and 15 older faces. These stimuli were selected from a larger face stimulus pool that had been developed and validated in prior stimulus-preparation work for a series of studies on age-related face perception. The original pool included 250 facial images from three age groups (80 younger, 85 middle-aged, and 85 older faces). Because the present study focused specifically on intergenerational decisions involving younger and older adults, only younger and older faces were selected for the current experiment. The original face images were collected from publicly available online sources and the Chicago Face Database (D. S. Ma et al., 2015). All faces were selected based on the following criteria: (1) neutral emotional expression, (2) front-facing orientation with direct gaze toward the camera, and (3) no glasses, jewelry, or other visible accessories. To standardize the stimuli, all face images were (1) normalized to inter-pupillary distance using algorithms provided by the OpenCV computer vision library (https://opencv.org/) and facial landmarks provided by the dlib machine learning toolkit (http://dlib.net/); (2) resized and cropped to 450 pixels (width) × 540 pixels (height); (3) placed onto a plain black background using the GIMP 2 software package (https://www.gimp.org/); and (4) color corrected (Workman et al., 2021; He et al., 2021).

A separate sample of 80 raters (67 males, 13 females; age: *M* = 19.95, *SD* = 2.21; education: *M* = 14.30, *SD* = 1.75), recruited from universities in South China, evaluated a random subset of 60 faces each on the following dimensions: perceived age category (e.g., young, middle-aged, or older), perceived age range (e.g., ≥60 years), estimated specific age, familiarity, emotion valence, and attractiveness. Ratings were collected via an online survey administered through Qualtrics (https://www.qualtrics.com). Based on the purpose of the present research, only younger and older faces were selected for use. A final set of 30 faces (15 younger, 15 older; 7 males and 8 females per group) were chosen according to the following criteria: (1) high consensus on age category classification (younger faces predominantly rated as “young person”; older faces as “older person”); (2) high consensus on age range (younger faces rated as 20–29 years; older faces as ≥60 years); (3) estimated specific ages falling within the expected range for each category; (4) no significant group differences in familiarity; and (5) balanced proportions of attractiveness levels within each group. Of the final 30 faces, 29 were sourced from the internet and 1 from the Chicago Face Database.

Independent-samples *t*-tests confirmed that the two groups of faces were well matched on familiarity, *t*(24.21) = −1.09, *p* > 0.05 (younger: *M* = 3.34, *SD* = 0.38; older: *M* = 3.15, *SD* = 0.58), and emotional valence, *t*(27.96) = −0.43, *p* > 0.05 (younger: M = 3.91, *SD* = 0.48; older: *M* = 3.83, *SD* = 0.50). As expected, the two groups differed significantly in perceived age, *t*(21.49) = 32.40, *p* < 0.001 (younger: *M* = 24.78, *SD* = 2.17; older: *M* = 63.06, *SD* = 4.03). Attractiveness was significantly higher for younger than older faces, *t*(25.86) = −3.85, *p* < 0.001, consistent with the well-documented age-related decline in perceived facial attractiveness (He et al., 2021). The face-rating questionnaire, raw data, analysis scripts, and final stimulus information are publicly available on the Open Science Framework (https://osf.io/wnxpm, accessed on 1 February 2026). Due to copyright and privacy protection considerations, the face images are not displayed in this manuscript.

#### 2.1.3. Procedures

##### Demographics and Baseline Measures

Participants first completed a questionnaire before the Dictator Game. The questionnaire consisted of three parts: demographic information, baseline psychological measures, and a perspective-taking imagination task. The questionnaire took approximately 10–15 min to complete. In the demographic section, participants reported their gender, age, educational level, and contact with older adults (e.g., frequency, co-residence, caregiving experience, and relationship quality).

Participants then completed two baseline psychological measures before the experimental manipulation. Explicit ageist attitudes were assessed using Kogan’s Attitudes Toward Older People Scale (Kogan OP; Kogan, 1961; Y.-E. Liu et al., 2014). The questionnaire included 26 items, one of which was an attention-check item. The attention-check item was excluded from scoring, resulting in 25 scored items. Participants rated each item on a 7-point Likert scale ranging from 1 = strongly disagree to 7 = strongly agree. Positive items were reverse-coded so that higher scores indicated more negative explicit attitudes toward older adults, that is, stronger explicit ageist attitudes. Cronbach’s α in the present sample was 0.90.

Dispositional empathy was assessed using the Interpersonal Reactivity Index (IRI; Davis, 1980). The questionnaire included 23 items, one of which was an attention-check item. The attention-check item was excluded from scoring, resulting in 22 scored items. Participants rated each item on a 5-point Likert scale ranging from 1 = does not describe me well to 5 = describes me very well. After reverse coding where appropriate, higher scores indicated higher levels of dispositional empathy. Cronbach’s α in the present sample was 0.87.

These baseline measures were administered to examine whether the two experimental conditions were comparable in explicit ageist attitudes and dispositional empathy before the manipulation.

##### Perspective-Taking Manipulation

The experimental manipulation was adapted from Engle-Friedman et al. (2022). Participants were randomly assigned to one of two conditions: (1) Future-aged-self condition: Participants imagined, drew, and wrote about themselves in their future elderly years (i.e., after age 60). (2) Current-self condition: Participants imagined, drew, and wrote about their current life. In both conditions, participants were asked to draw a picture on paper of the person identified in that condition and to write about that person, including family life, living arrangements, how most of their time was spent, and what the person did for fun in their free time. This manipulation served as a perspective-taking induction, encouraging vivid simulation of either the present or future self. The task structure followed established procedures in perspective-taking interventions (Galinsky & Moskowitz, 2000; Oh et al., 2016).

##### Manipulation Check

**Figure 1:**
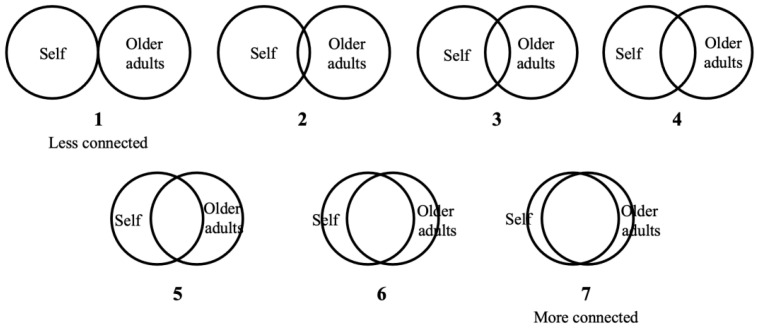
The inclusion of other in the self scale.

After the imagination task, participants completed the Inclusion of Other in the Self (IOS) Scale (Aron et al., 2004) to assess perceived closeness between the self and older adults. They viewed seven pairs of overlapping circles (Figure 1), with one circle representing themselves and the other representing older adults, including their future aged self. Participants selected the pair that best reflected their perceived closeness. Higher scores indicated greater self–other overlap with older adults.

##### Dictator Game

Finally, participants completed a modified Dictator Game (DG) adapted from Workman et al. (2021). The DG is a widely used measure of prosocial behaviors such as fairness and altruism (Ben-Ner et al., 2009). Each participant acted as the “dictator” in 15 rounds, deciding how to allocate ¥5 between themselves and another player represented by a facial photograph. Before the task began, participants were informed that the face photographs represented real individuals who had also participated in the study, and that their allocation decisions would influence the actual participation payment those individuals received. This instruction aimed to enhance ecological validity and strengthen participants’ sense of moral responsibility. Photographs depicted younger or older faces (7–8 trials per category). In each trial, participants chose between two randomly selected splits, ranging from keeping the entire ¥5 to sharing it 50:50. The split entailing the smallest difference in payoffs between dictator and receiver was considered prosocial. The experiment was programmed using OpenSesame 3.3.9, with all trials presented in randomized order. Participants proceeded at their own pace without time limits.

##### Debriefing

Upon completion, participants were debriefed as to the purpose of this study.

#### 2.1.4. Data Analyses

Linear mixed-effects analyses were carried out using the lme4 package (Bates et al., 2015) in R 4.3.2 (R Core Team, 2023) to examine how group (future-aged-self vs. current-self) and face age (younger vs. older) influenced young adults’ prosocial behavior toward younger and older people. We obtained p values for the parameter estimates generated by each model using Satterthwaite’s approximation as implemented by the lmerTest package (Kuznetsova et al., 2017). Post hoc pairwise comparisons were conducted with the lsmeans package (Lenth, 2016). Below, regression coefficients (β), standard errors (*SE*), and *t*-values are reported. Partial experimental materials, data, and analysis scripts for both studies are publicly available on the Open Science Framework (https://osf.io/wnxpm, accessed on 1 February 2026).

### 2.2. Results and Discussion

#### 2.2.1. Manipulation Check: IOS

To verify the effectiveness of the experimental manipulation, a linear model compared self–other overlap scores between groups. Participants who imagined their future aged self reported significantly higher IOS scores (*M* = 4.04, *SD* = 1.00) than those who imagined their current self (*M* = 3.01, *SD* = 1.35), *t*(158) = 5.45, *p* < 0.001. This indicates that the perspective-taking manipulation successfully increased perceived overlap with older adults.

#### 2.2.2. Baseline Equivalence: Kogan OP and IRI

As a baseline equivalence check, we compared the two experimental conditions on pre-manipulation measures of explicit attitudes toward older adults and dispositional empathy. No significant group differences were observed for Kogan OP or IRI scores, all *p*s > 0.10. These results suggest that the two conditions were comparable in baseline attitudes toward older adults and trait empathy before the manipulation.

#### 2.2.3. Dictator Game

A linear mixed-effects model examined whether group (future-aged-self vs. current-self) and face age (younger vs. older) influenced the amount of money allocated to recipients. The dependent variable was the amount of money allocated to the recipient, with random intercepts for participants. Significant main effects were observed for both group and face age. Participants in the future-aged-self group allocated more money overall than those in the current-self group (*p* < 0.001), and participants allocated more money to older faces than to younger faces overall (*p* < 0.001; see Table 1 and Table 2).

**Figure 2:**
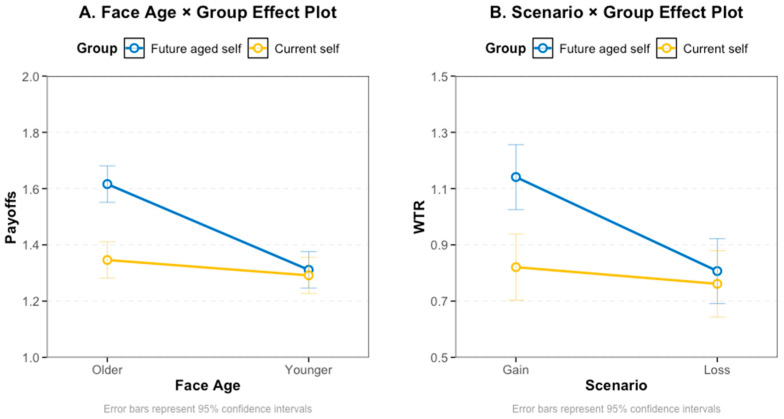
(**A**) Interaction between group and face age on payoffs in Study 1; (**B**) interaction between group and scenario framing on WTR in Study 2.

For prosocial behavior assessed with the Dictator Game, there was a significant interaction between face age and group on allocation amount (β = 0.25, *SE* = 0.054, 95% CI [0.14, 0.36], *t*(2247.9) = 4.603, *p* < 0.001 (see Table 1 and Table 2, Figure 2). To better understand this interaction, we conducted post hoc pairwise comparisons interrogating this interaction. For older faces, participants in the future-aged-self group allocated significantly more money than those in the current-self group, *t*(356) = 5.76, *p* < 0.001, d = 0.41, 95% CI [0.27, 0.55]. Within the future-aged-self group, allocations to older faces were significantly higher than to younger faces, *t*(2248) = 7.94, *p* < 0.001, d = 0.46, 95% CI [0.34, 0.57]. No significant difference was observed between young and old faces in the current-self group (*t*(2248) = 1.43, *p* > 0.10, d = 0.08), nor between groups for younger faces (*t*(350) = 0.42, *p* > 0.50, d = 0.03).

These findings indicate that imagining one’s future aged self enhanced prosocial allocation toward older adults. Together with the manipulation-check result, this pattern is consistent with the view that future-aged-self perspective taking increased psychological closeness to older adults and promoted prosocial behavior toward this group. Together with the manipulation-check result, this pattern is consistent with the possibility that increased psychological closeness to older adults contributed to greater prosocial allocation toward them.

Although Study 1 showed that imagining one’s future aged self increased self–other overlap and promoted more generous allocations to older adults, these effects may not be universal. Importantly, the Dictator Game resembles a gain-framed context in which helping older adults primarily involved foregoing potential gains rather than making decisions explicitly framed as personal losses. As such, the task does not capture situations in which helping entails explicit personal costs, which is common in real-world intergenerational decisions involving limited resources (e.g., pensions, healthcare, employment opportunities). As outlined in the introduction, loss-framed or resource-competitive contexts may activate loss aversion and self-protective motives (Sherif, 1966; Kahneman & Tversky, 1979), potentially limiting the prosocial impact of perspective-taking interventions. To address this limitation, Study 2 incorporated both gain- and loss-framed scenarios using the Welfare Trade-Off Task (WTT; Delton, 2010; Kirkpatrick et al., 2015), enabling a more precise examination of how individuals balance their own welfare against that of older adults when helping either yields additional benefits (gain frame) or is framed as imposing potential personal costs (loss frame).

Moreover, Study 2 strengthened the manipulation by incorporating a personalized, age-progressed image of each participant’s own face. Compared with imagination alone, visually confronting one’s aged self may heighten future-self continuity and make the older-adult identity more self-relevant (Hershfield et al., 2012; Van Gelder et al., 2013). This design allowed for a more rigorous examination of our second hypothesis.

## 3. Study 2

### 3.1. Materials and Methods

#### 3.1.1. Participants

A total of 143 participants were recruited from South China Normal University and Guangdong University of Education (102 females; age: *M* = 20.81, *SD* = 3.05, range: 18–33 years; education: *M* = 15.03, *SD* = 2.09). As in Study 1, an a priori power analysis (f = 0.25, α = 0.05, power = 0.95) indicated that a minimum of 54 participants was required. Participants were randomly assigned to one of two groups: (1) Future-aged-self condition, *n* = 73, 53 females; age: *M* = 20.62, *SD* = 3.09; education: *M* = 14.92, *SD* = 2.17. (2) Current-self condition, *n* = 70, 49 females; age: *M* = 21.01, *SD* = 3.01; education: *M* = 15.14, *SD* = 2.00. All participants provided written informed consent before participating, including consent for the use of their facial images for research purposes, and received 20 RMB as compensation after completing the experiment. The study protocol was approved by the Institutional Review Board of Guangdong University of Education. Data collection was conducted between September and December 2024.

#### 3.1.2. Materials

**Figure 3:**
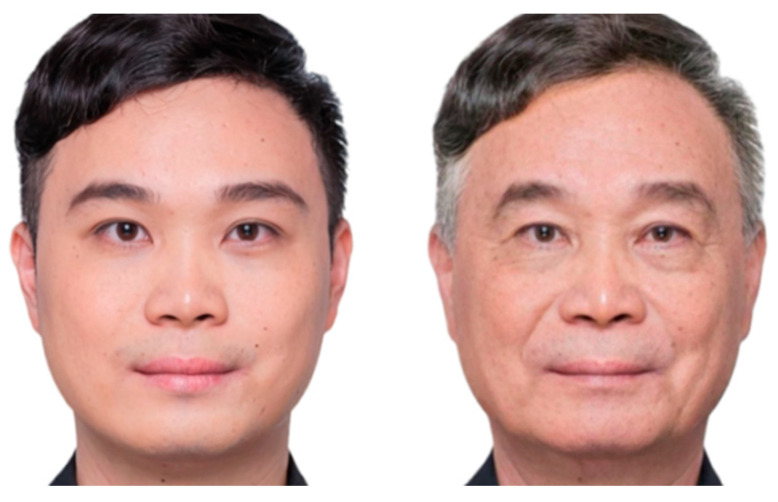
Sample stimuli of the Study 2 perspective-taking manipulation. The individual depicted in this figure provided written informed consent for the publication of his image.

In the perspective-taking manipulation, the stimulus set comprised 143 facial images: 70 unaltered photographs from participants in the current-self condition and 73 age-progressed images generated from participants in the future-aged-self condition (see Figure 3 for examples). Prior to the experiment, all individuals provided a recent neutral-expression photograph and granted written consent for its use. Age-progressed stimuli depicting later-life appearance were created using FaceApp (https://www.faceapp.com) based on participants’ photographs in the future-aged-self condition.

In the Welfare Trade-Off Task, the stimuli consisted of 20 older faces selected from the validated face-stimulus pool described in Study 1, including the older faces used in Study 1 and additional older faces meeting the same validation criteria.

#### 3.1.3. Procedures

##### Demographics and Baseline Measures

Before the manipulation, participants completed demographic questions and the same baseline psychological measures used in Study 1. Cronbach’s α values in the present sample were 0.88 for the Kogan OP and 0.86 for the IRI. Additionally, an age-related Implicit Association Test (IAT) was administered to assess baseline implicit attitudes toward older adults. These measures were used to examine whether the two experimental conditions were comparable before the manipulation.

##### Perspective-Taking Manipulation

The experimental manipulation was adapted from prior future-self and aging-simulation research (Engle-Friedman et al., 2022; Van Gelder et al., 2013; Oh et al., 2016). Participants were randomly assigned to either the future-aged-self condition or the current-self condition.

In the future-aged-self condition, participants viewed a computer-generated image of their future aged self. They were instructed to carefully observe the image and imagine themselves in later life, defined as age 60 or older. Participants first rated the perceived realism of the image on a 7-point scale ranging from 1 = very unrealistic to 7 = very realistic. They were then asked to imagine and write about four aspects of their future older life: family life, work life, typical daily routine, and possible difficulties they might encounter in later life.

In the current-self condition, participants viewed a current-self image and were instructed to carefully observe the image and imagine their current daily life. Participants first rated the extent to which the image matched their current appearance on a 7-point scale ranging from 1 = does not match me at all to 7 = matches me very well. They were then asked to imagine and write about four parallel aspects of their current life: family life, work or study life, typical daily routine, and possible difficulties they might currently encounter.

This manipulation was designed to encourage vivid simulation of either the future aged self or the current self while keeping the structure of the writing task parallel across conditions.

##### Manipulation Check

Both groups then completed the IOS Scale (Aron et al., 2004) to report their perceived self–other overlap with older adults.

##### Future Contact Intention

Contact intention toward older adults was assessed using three items adapted from prior work (e.g., Oh et al., 2016). Participants indicated their agreement with the following statements on a 7-point Likert scale (1 = strongly disagree, 7 = strongly agree): (1) “I intend to interact frequently with older adults”; (2) “I plan to learn more about older adults”; (3) “I believe it is important to interact with older adults in the future.” Higher scores indicated stronger intentions to communicate with older adults in the future.

##### Welfare Trade-Off Task

**Figure 4:**
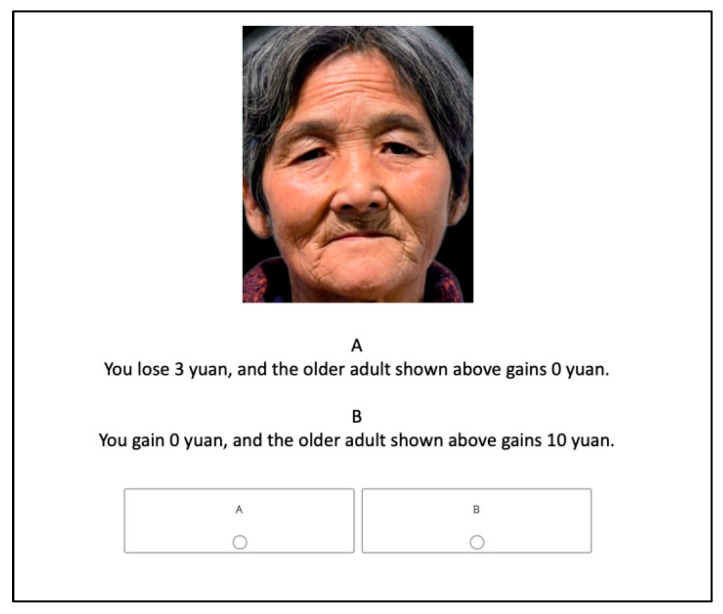
Example trial in the WTT.

Finally, participants completed a Welfare Trade-Off Task (WTT), adapted from Kirkpatrick et al. (2015) and Gao et al. (2020), in which they made a series of forced-choice decisions between monetary outcomes for themselves versus another person. On each trial, participants viewed a facial stimulus and then selected one of two allocation options (Figure 4). Depending on the scenario, choices involved either gains or losses for the self and the other party.

In the gain frame, participants chose between a selfish option (self gains: ¥15 to −¥3; other: ¥0) and a prosocial option (self: ¥0; other: + ¥10). In the loss frame, they chose between a prosocial option (self losses: −¥15 to −¥3; other: ¥0) and a selfish option (self: ¥0; other: −¥10). See Table 3 for the details. Participants were informed that the face images represented real study participants and that a subset of decisions would influence final bonus payments, thereby increasing ecological validity. Following practice trials, participants completed the formal task. To counterbalance order effects, half of the participants completed the gain block first and the loss block second, whereas the other half completed the blocks in the reverse order. The task lasted approximately 10 min.

The subjective indifference point, operationalized as the welfare trade-off ratio (WTR), served as the primary prosociality index. WTR was calculated as follows: (1) The self-to-other payoff ratio was computed for each trial (see Table 3). (2) Identify the first trial in which the participant switched from the selfish to the prosocial option (gain frame) or vice versa (loss frame). (3) Compute the WTR as the average of the ratio at the switching trial and the preceding ratio. Participants who consistently chose the prosocial option were assigned the maximum WTR value (1.60), as in prior work (Gao et al., 2020; Kirkpatrick et al., 2015).

Consistent with Kirkpatrick et al. (2015) and Gao et al. (2020), the WTR ranged from −0.20 to 1.60, reflecting decisions from highly selfish to highly altruistic. At the selfish extreme, participants preferred losing ¥3 rather than allowing others to gain ¥10, or gaining ¥3 while imposing a ¥10 loss on others. At the altruistic extreme, participants were willing to sacrifice a ¥15 gain to grant others ¥10, or lose ¥15 to prevent others from losing ¥10. Pilot testing also showed that ¥15 is a meaningful and difficult-to-forgo amount for college students; thus, the task’s maximum self-gain/loss was set at ¥15. The experiment was conducted in person via Qualtrics.

##### Debriefing

Upon completion, participants were debriefed as to the purpose of this study.

#### 3.1.4. Data Analyses

The analytical approach was identical to Study 1. Linear mixed-effects models were fitted with WTR as the dependent variable, group and scenario as fixed factors, and random intercepts for participants.

### 3.2. Results and Discussion

#### 3.2.1. Manipulation Check: IOS and Future Contact Intention

To verify the effectiveness of the manipulation, we first compared self–other overlap scores between groups using the IOS scale. Participants in the future-aged-self group (*M* = 4.16, *SD* = 1.05) reported significantly higher self–other overlap with older adults than those in the current-self group (*M* = 3.04, *SD* = 1.46), *t*(141) = 5.29, *p* < 0.001. We also assessed future communication intentions. Similarly, participants in the future-aged-self group expressed a significantly stronger intention (*M* = 4.95, *SD* = 0.82) to engage with older adults than those in the current-self group (*M* = 4.31, *SD* = 1.08), *t*(141) = 4.00, *p* < 0.001. These findings confirm that future-aged-self perspective-taking manipulation successfully increased psychological closeness to older adults and willingness to communicate with them.

#### 3.2.2. Baseline Equivalence: Kogan OP, IRI, and IAT

As a baseline equivalence check, we compared the two experimental conditions on pre-manipulation measures of explicit ageist attitudes, dispositional empathy, and implicit age bias. No significant group differences were observed for Kogan OP, IRI, or IAT scores, all *p*s > 0.10. These results suggest that the two conditions were comparable in baseline explicit ageist attitudes, trait empathy, and implicit age bias before the manipulation.

#### 3.2.3. Welfare Trade-Off Task

A linear mixed-effects model was conducted with WTR as the dependent variable, group (Future-aged-self vs. Current-self) and scenario (Gain vs. Loss) as fixed factors, and a random intercept for participants. Significant main effects were observed for both group and scenario. The future-aged-self group showed higher WTR than the current-self group, *p* < 0.001, indicating stronger willingness to prioritize older adults’ welfare (see Table 4 and Table 5). WTRs were significantly higher in the gain context than in the loss context, *p* < 0.001, consistent with loss-aversion literature.

A significant interaction effect between group and scenario was observed, β = 0.27, *SE* = 0.07, 95% CI [0.13, 0.42], *t*(141) = 3.74, *p* < 0.001, indicating that the effect of perspective taking varied across contexts (see Table 4 and Figure 2). Post hoc analyses clarified this pattern (see Table 5). In the gain scenario, participants in the future-aged-self group showed a significantly higher WTR compared with the current-self group, *t*(204) = 3.82, *p* < 0.001, d = 1.03, 95% CI [0.49, 1.57]. The current-self group showed a mean WTR of 0.82, suggesting that participants were generally willing to forgo approximately 6–8 RMB of their own payoff to allocate 10 RMB to the target (see Table 3 and Table 5). The future-aged-self group showed a significantly higher mean WTR of 1.14, suggesting they were willing to sacrifice about 10 RMB of personal gain to provide 10 RMB to the other person. This pattern suggests that when helping others involved giving up potential gains, future-aged-self perspective taking increased welfare valuation of older adults. Together with the IOS results, this pattern is consistent with the possibility that increased psychological closeness contributed to greater prosocial responding.

In the loss scenario, however, no significant group differences were observed, *t*(204) = 0.54, *p* > 0.50, d = 0.15, 95% CI [−0.39, 0.68]. Participants in both conditions were similarly willing to forgo approximately 6–8 RMB to avoid deducting 10 RMB from the older target. As outlined in the introduction, loss-framed contexts tend to elicit stronger self-protective motivations and may reduce the influence of perspective-taking interventions. In the WTT, decisions in the loss frame required participants to consider accepting potential monetary losses, which likely activated loss aversion and a focus on avoiding personal harm. Under these motivational pressures, the prosocial benefits of imagining one’s future aged self may have been constrained, which could help explain why group differences observed in gain-framed scenarios did not emerge under loss frames.

## 4. General Discussion

Population aging has brought unprecedented social, economic, and public-health challenges, making ageism a pressing global concern. Although many studies have examined the content of age-related prejudice, its consequences, and intervention strategies (e.g., Burnes et al., 2019; Cuddy et al., 2005; Kite et al., 2005; Levy, 2009; North & Fiske, 2012, 2015; Spaccatini et al., 2022), far fewer have leveraged ageism’s unique temporal-self dimension. Drawing on Stereotype Embodiment Theory (Levy, 2009) and future-self continuity frameworks (Engle-Friedman et al., 2022; Hershfield et al., 2012), the present research investigated whether imagining one’s future aged self can increase perceived psychological closeness to older adults and promote prosocial behavior toward them, and whether such effects depend on the decision-making context. We found that (1) the manipulation reliably increased self–other overlap with older adults (IOS); (2) it promoted prosocial behavioral responses (Study 1: higher allocations to older adults in a Dictator Game; Study 2: elevated WTR in gain-framed contexts and stronger intentions for future communication), yet (3) in loss-framed contexts the prosocial advantage attenuated to non-significance (Study 2). Importantly, these effects were most directly reflected in perceived psychological closeness and prosocial outcomes, including future communication intentions and behavioral resource allocation, rather than in direct evidence of broad attitudinal change. These findings indicate that future-self perspective taking can enhance prosocial behavioral responses toward older adults, but its efficacy is context-specific rather than universal, highlighting both the promise of temporal-self engagement and the importance of contextual boundary conditions.

### 4.1. Possible Psychological Pathways Linking the Self to Ageism-Related Prosocial Outcomes

Across both studies, engaging with one’s future aged self consistently increased perceived closeness to older adults and promoted prosociality. Study 1 showed that future-aged-self perspective taking was associated with greater generosity in a Dictator Game. Participants in the future-aged-self condition allocated more money to older (vs. younger) targets. Study 2 extended this pattern by showing that future-aged-self perspective taking increased WTRs toward older adults in gain-framed contexts. These findings are consistent with previous evidence that greater inclusion of others in the self is associated with heightened empathy, cooperation, ethical behavior, and altruism (Bernstein et al., 2015; Cialdini et al., 1997; Hershfield et al., 2012; Zhong et al., 2015). Related VR research similarly shows that embodying an aged avatar can increase intentions to interact with older adults (Oh et al., 2016). Together, these findings suggest that increasing psychological connectedness with older adults may be a promising route to enhancing prosocial responding toward this group.

These results are consistent with the idea that age-related biases in prosocial decision-making may be reduced by softening the psychological boundary between the self and older adults. Social Identity Theory (Tajfel & Turner, 1979, 1986) and social categorization theory (Brown, 2011) suggest that biases emerge, in part, when older adults are categorized as an outgroup (“them”). By imagining their own future aged selves, participants may have temporarily blurred this categorical boundary, shifting older adults psychologically closer to “us.” This interpretation also aligns with evidence that ageism is partly driven by symbolic threats to youth identity. Individuals who strongly identify as young tend to perceive older adults as threatening to their vitality and self-image, which can amplify derogation and social distance (North & Fiske, 2012; Shimizu & Grassi, 2022; Shimizu et al., 2022). Prior evidence further shows that when older adults are excluded from younger adults’ self-group, younger individuals tend to show less helping and more hostile ageism toward them (Chen & Zhang, 2022; G. Ma et al., 2024; Tasdemir, 2020). By prompting participants to envision themselves as future members of the very group from which they may otherwise distance themselves, the manipulation may have reduced this perceived symbolic threat and weakened the defensive motivation to maintain a positive youth-based identity. The increased self–other overlap observed in our studies is consistent with this possibility, suggesting that future-self perspective taking may soften the psychological boundary separating “young” from “old” and thereby facilitate greater prosocial responding toward older adults.

Our findings also support the temporal-self perspective emphasized in Stereotype Embodiment Theory (SET; Levy, 2009). As discussed earlier, ageism is unique because the “outgroup” of today becomes the “future self” of tomorrow. SET-based interventions show that making this inevitability salient, by highlighting how internalized stereotypes ultimately harm one’s future self, can activate both altruistic motives toward older adults and self-protective motives aimed at avoiding future disadvantage (Shimizu & Grassi, 2022; Shimizu et al., 2022). In a similar way, imagining one’s future aged self in the present study may have increased perceived temporal continuity and made older adults more self-relevant, encouraging participants to treat older adults less as a distant outgroup.

Finally, this pattern also aligns with Self-Expansion Theory (Aron et al., 2013), which proposes that individuals may broaden their self-concept by incorporating others’ attributes, perspectives, and welfare into the self. Prior research shows that visualizing or embodying one’s future self increases psychological closeness, moral restraint, and prosocial orientation (Engle-Friedman et al., 2022; Hershfield et al., 2012; Van Gelder et al., 2013). This is also consistent with the idea that psychological connectedness motivates individuals to protect the interests of their future selves (Bartels & Rips, 2010). In the present research, future-aged-self perspective taking may have expanded the self-boundaries to include older adults as representatives of one’s future identity. When older adults become more psychologically connected to the self, their welfare may become more self-relevant, which could increase generosity and willingness to sacrifice in gain-framed decisions. However, because empathic concern and future-self continuity were not directly measured as post-manipulation process variables, this interpretation should be treated as a plausible pathway rather than a demonstrated mechanism.

Taken together, these patterns are consistent with an integrative pathway in which imagining one’s future aged self may promote ageism-related prosocial outcomes by increasing perceived temporal continuity with one’s future self, softening categorical boundaries between “young” and “old,” and expanding the psychological self to include older adults. Through these combined processes, older adults’ outcomes may become more psychologically self-relevant, thereby potentially enhancing the weight placed on their welfare in prosocial decisions.

### 4.2. Contextual Moderators of Future-Self Interventions

However, the prosocial effect of future-aged-self perspective taking was not universal. In the WTT, the group difference observed in gain-framed scenarios did not emerge in loss-framed scenarios. When helping was framed as involving potential personal costs, perspective taking did not produce a statistically detectable increase in prosocial welfare trade-offs. This pattern suggests that motivational forces associated with loss contexts may attenuate the benefits of heightened psychological closeness. It also contributes to research on distributive decision-making by showing that the same future-self manipulation can have different behavioral consequences depending on whether the decision is framed as allocating potential gains or accepting potential losses. Our findings are consistent with growing evidence that interventions aimed at enhancing social preferences, including empathy and perspective taking, often produce reliable effects in low-cost laboratory settings but yield more modest, context-dependent outcomes when resource conflict or costly behavior is involved (Koessler et al., 2023; Levitt & List, 2007; Ortiz-Riomalo et al., 2021; Sassenrath et al., 2022; Vorauer, 2013).

One plausible explanation derives from Prospect Theory, which proposes that losses loom larger than equivalent gains and therefore elicit stronger self-protective motives (Kahneman & Tversky, 1979). Although our manipulation increased self–other overlap, loss-framed decisions may have made potential personal costs more salient, thereby reducing participants’ willingness to sacrifice their own welfare for older adults. In this sense, psychological closeness may facilitate prosociality when helping is framed as allocating potential gains, but its influence may be constrained when helping is framed as preventing losses at a personal cost.

A complementary interpretation comes from threat-based accounts of intergroup relations. Integrated Threat Theory (Stephan & Stephan, 2000) proposes that perceived threats to the ingroup’s welfare can trigger anxiety and defensive responses toward outgroups. Similarly, Realistic Group Conflict Theory (Sherif, 1966) suggests that when resources are perceived as scarce or zero-sum, individuals may respond with heightened defensiveness and reduced altruism. In the present study, loss-framed WTT scenarios may have made the potential cost to the self more salient, thereby limiting the extent to which increased closeness to older adults translated into greater welfare allocation. This interpretation is consistent with work on aging attitudes showing that public debates over pensions, health care, and employment can evoke zero-sum beliefs and intensify age-based tension (North & Fiske, 2013).

These dynamics can also be interpreted within the welfare trade-off ratio framework. Prior research suggests that individuals dynamically adjust the weight placed on others’ welfare as a function of psychological distance, perceived threat, and reference points (Delton & Robertson, 2016; Gao et al., 2020; Hall et al., 2021; Kirkpatrick et al., 2015). Our findings suggest that future-aged-self perspective taking may increase WTRs toward older adults in gain-framed contexts, whereas loss-framed contexts may limit the behavioral expression of this increased psychological closeness. Thus, the WTR framework helps clarify why the same perspective-taking manipulation may produce different behavioral effects depending on whether the decision context emphasizes gains or losses.

Overall, the two studies jointly show that temporal-self engagement can raise younger adults’ willingness to prioritize older adults’ welfare, as reflected in increased self–other overlap, stronger future contact intentions, and more generous resource allocations. However, this effect is bounded by the gain–loss structure of the decision, which is precisely the type of contextual moderation highlighted in our theoretical framework.

### 4.3. Theoretical and Practical Implications

#### 4.3.1. Theoretical Implications

The present research makes two primary contributions beyond previous work. First, we extend the literature on perspective taking and ageism by explicitly incorporating a temporal-self component that has been largely overlooked in prior interventions. By asking young adults to imagine their future aged selves, and in Study 2 to view an age-progressed rendering of their own face, our research directly targets the temporal nature of ageism and examines self–other overlap as a theoretically grounded indicator of psychological closeness to older adults. Second, by integrating this manipulation with the Welfare Trade-Off Task, we demonstrate that the prosocial effects of future-self perspective taking are context-dependent rather than universal. This boundary condition advances a more nuanced understanding of when and how perspective taking can effectively reduce ageism.

#### 4.3.2. Practical Implications

Our research suggests that temporal-self processes may play an important role in shaping intergenerational attitudes and decisions. Interventions aimed at reducing ageism may benefit from pairing future-self perspective taking with structural reframing. In applied settings, such as intergenerational programs, healthcare triage attitudes, or workplace succession planning, designing choice contexts that highlight mutual gains rather than zero-sum trade-offs, emphasize long-term benefits to one’s future self, or smooth perceived losses (e.g., through phased resource shifts) may help sustain prosocial responses toward older adults. Such layered approaches could enhance the impact of future-self interventions, particularly when intergenerational decisions involve potential costs.

### 4.4. Limitations and Future Directions

Several limitations warrant attention. First, the samples consisted primarily of young Chinese university students, which limits the generalizability of the findings across age groups, cultural contexts, and educational backgrounds. Middle-aged and older adults may respond differently to future-self manipulations, particularly because the psychological distance between the present self and the older self may vary across the life span. Future research should examine whether the present findings replicate in more diverse samples, including middle-aged adults, older adults, non-university populations, and participants from cultural contexts in which intergenerational norms and obligations may differ.

Second, the manipulations were brief laboratory-based inductions, and the behavioral tasks were conducted in controlled experimental settings. Future research should examine the durability of these effects and compare different intervention formats, such as repeated future-self reflection, immersive virtual reality, or intergenerational contact combined with future-self perspective taking. Field studies involving real intergenerational decisions would also help assess the ecological validity of the findings.

Third, although IOS and gain–loss framing provided theoretically relevant evidence regarding psychological closeness and contextual boundary conditions, the current design does not permit formal mediation testing. Future research should incorporate direct process measures such as empathic concern, future-self continuity, zero-sum beliefs, and perceived symbolic threat, and formally test moderated mediation models, such as perspective taking → self–other overlap → empathy → WTR, moderated by gain–loss framing, to clarify how and when future-self engagement translates into prosocial behavior across different decision contexts.

Finally, neither study included filler or interference tasks designed to obscure the purpose of the experiment. Given the transparent link between the future-aged-self imagination task and the subsequent measures involving older adult targets, participants may have inferred the study’s purpose and adjusted their responses accordingly, a concern commonly referred to as demand characteristics (Orne, 1962). Although the condition-specific patterns observed in Study 1 and Study 2 suggest that responses were not uniformly elevated across all outcomes or contexts, we cannot rule out the possibility that some responses were influenced by perceived experimenter expectations. Future studies should consider incorporating filler tasks, cover stories, suspicion probes, or delayed behavioral outcomes to reduce the salience of the study’s objectives and better assess possible demand effects.

Theoretically, these limitations suggest that future work on ageism should distinguish more clearly between changes in ageist attitudes, psychological closeness to older adults, and ageism-related prosocial behavior. The present findings provide stronger evidence for the latter two outcomes than for broad attitudinal change, and future studies should examine whether temporal-self interventions can produce durable changes across multiple components of ageism.

Despite these limitations, the present research provides a theoretically grounded starting point for understanding when and how future-self engagement can promote prosocial responding toward older adults and contribute to intergenerational cohesion.

## 5. Conclusions

The present research examined how imagining one’s future aged self shapes psychological closeness and prosocial responses toward older adults. Across two studies, future-aged-self perspective taking reliably increased self–other overlap with older adults and promoted prosocial responding, including more generous resource allocations and stronger future contact intentions, particularly in gain-framed situations. However, these effects diminished when helping was framed as involving potential personal costs. These findings suggest that reducing age-related bias in intergenerational decision-making may depend not only on increasing psychological connectedness between age groups but also on the broader context in which such decisions are made. Combining temporal-self interventions with gain-oriented framing may offer a promising approach for fostering intergenerational understanding and cooperation.

## Figures and Tables

**Table 1:** Fixed effects from the linear mixed-effects model examining how the amount of money allocated varied as a function of group and face age.

Fixed Effects	β	*SE*	*t* Value	*p* Value
Intercept	1.62	0.03	48.77	<0.001
Face age (Younger)	−0.30	0.04	−7.94	<0.001
Group (Control)	−0.27	0.05	−5.76	<0.001
Face age (Younger): Group (Control)	0.25	0.05	4.60	<0.001

Note. *SE*, standard error. The Intercept represents the estimated mean allocation for older faces in the future-aged-self group. The reference levels are “Older face” for face age and “Future-aged-self” for Group. “Face age (Younger): Group (Control)” represents the interaction effect, testing whether the effect of face age on allocations differs between the two groups.

**Table 2:** Means and standard deviations of the amount of money allocated in the Dictator Game by group and face age.

	Younger Face	Older Face	Overall
Future aged self	1.31 (0.71)	1.62 (0.62)	1.46 (0.69)
Current self	1.29 (0.69)	1.34 (0.72)	1.32 (0.70)
Overall	1.30 (0.70)	1.48 (0.69)	

**Table 3:** Trial structure and corresponding WTR values across gain and loss conditions.

Trial	Option	Self Gain (Loss)	Other Gain (Loss) ^1^	Self–Other Benefit Ratio	WTR
1	A B	150	010	1.50	1.6
2	A B	120	010	1.20	1.35
3	A B	100	010	1.00	1.10
4	A B	80	010	0.80	0.90
5	A B	60	010	0.60	0.70
6	A B	40	010	0.40	0.50
7	A B	20	010	0.20	0.30
8	A B	10	010	0.10	0.15
9	A B	−10	010	−0.10	0.00
10	A B	−30	010	−0.30	−0.20

^1^ Other gain (loss) refers to increases (decreases) in monetary payoff for the older target whose face was presented in the WTT.

**Table 4:** Fixed effects from the linear mixed models constructed to examine how the WTR varies by group and scenario.

Fixed Effects	β	*SE*	*t* Value	*p* Value
Intercept	1.14	0.06	19.46	<0.001
Scenario (Loss)	−0.33	0.05	−6.50	<0.001
Group (Control)	−0.32	0.08	−3.82	<0.001
Scenario (Loss): Group (Control)	0.27	0.07	3.74	<0.001

Note. *SE*, standard error. The Intercept represents the estimated mean WTR in the gain scenario for the future-aged-self group. The reference levels are “Gain” for Scenario and “Future-aged-self” for Group. “Scenario (Loss): Group (Control)” represents the interaction effect, testing whether the effect of scenario on WTR differs between the two groups.

**Table 5:** Means and standard deviations of WTR by condition and scenario.

Group	Gain	Loss	Overall
Future-aged-self	1.14 (0.43)	0.81 (0.50)	0.97 (0.50)
Current-self	0.82 (0.57)	0.76 (0.50)	0.79 (0.53)
Overall	0.98 (0.53)	0.78 (0.50)	

## Data Availability

The datasets generated and analyzed during the current study are available from the corresponding author upon reasonable request.
